# Active Trachoma and Ocular *Chlamydia trachomatis* Infection in Two Gambian Regions: On Course for Elimination by 2020?

**DOI:** 10.1371/journal.pntd.0000573

**Published:** 2009-12-22

**Authors:** Emma M. Harding-Esch, Tansy Edwards, Ansumana Sillah, Isatou Sarr, Chrissy H. Roberts, Paul Snell, Esther Aryee, Sandra Molina, Martin J. Holland, David C. W. Mabey, Robin L. Bailey

**Affiliations:** 1 London School of Hygiene and Tropical Medicine, London, United Kingdom; 2 National Eye Care Programme, Ministry of Health, Banjul, The Gambia; 3 Medical Research Council Laboratories, Fajara, Banjul, The Gambia; 4 ALSPAC, Department of Social Medicine, University of Bristol, Bristol, United Kingdom; University of Cambridge, United Kingdom

## Abstract

**Background:**

Trachoma has been endemic in The Gambia for decades. National trachoma control activities have been in place since the mid-1980's, but with no mass antibiotic treatment campaign. We aimed to assess the prevalence of active trachoma and of actual ocular *Chlamydia trachomatis* infection as measured by polymerase chain reaction (PCR) in the two Gambian regions that had had the highest prevalence of trachoma in the last national survey in 1996 prior to planned national mass antibiotic treatment distribution in 2006.

**Methodology/Principal Findings:**

Two stage random sampling survey in 61 randomly selected Enumeration Areas (EAs) in North Bank Region (NBR) and Lower River Region (LRR). Fifty randomly selected children aged under 10 years were examined per EA for clinical signs of trachoma. In LRR, swabs were taken to test for ocular *C. trachomatis* infection. Unadjusted prevalences of active trachoma were calculated, as would be done in a trachoma control programme. The prevalence of trachomatous inflammation, follicular (TF) in the 2777 children aged 1–9 years was 12.3% (95% CI 8.8%–17.0%) in LRR and 10.0% (95% CI 7.7%–13.0%) in NBR, with significant variation within divisions (p<0.01), and a design effect of 3.474. Infection with *C. trachomatis* was found in only 0.3% (3/940) of children in LRR.

**Conclusions/Significance:**

This study shows a large discrepancy between the prevalence of trachoma clinical signs and ocular *C. trachomatis* infection in two Gambian regions. Assessment of trachoma based on clinical signs alone may lead to unnecessary treatment, since the prevalence of active trachoma remains high but *C. trachomatis* infection has all but disappeared. Assuming that repeated infection is required for progression to blinding sequelae, blinding trachoma is on course for elimination by 2020 in The Gambia.

## Author Summary

Trachoma is the leading infectious cause of blindness worldwide, and is mainly found in tropical and poor countries. It is caused by infection of the eyes with the bacterium *Chlamydia trachomatis*. However, sometimes the clinical signs of disease can be present without infection being detected. Control efforts involve surgery, antibiotic treatment, face washing, and environmental improvement for better hygiene. Surveys of trachoma help countries to know whether and where they should implement control interventions. The Gambia is found in West Africa and has suffered from trachoma for decades. We conducted a survey of two Gambian regions to look at how much trachoma disease and *C. trachomatis* infection there is in the eyes. We found that although there was enough disease (≥10%) to warrant antibiotic treatment for everyone in the regions, there was nearly no infection (0.3%). This means that using clinical signs alone to make treatment decisions in low prevalence settings like The Gambia can lead to the waste of scarce resources. Our results also suggest that since less than 1% of children are infected with *C. trachomatis*, The Gambia is on course to achieve the World Health Organization's aim of eliminating blinding trachoma by the year 2020.

## Introduction

Trachoma is the leading infectious cause of blindness worldwide.[1] It is caused by repeated re-infection with the ocular serotypes (A, B, Ba and C) of the bacterium *Chlamydia trachomatis*, and is predominantly found in the poorest countries in the world. Active trachoma, characterised by the presence of subepithelial follicles (trachomatous inflammation, follicular (TF)) and/or inflammation (trachomatous inflammation, intense (TI)), is usually found in children. After years of repeated re-infection scarring may occur, which can lead to distortion of the eyelid, causing the eyelashes to turn inwards (trichiasis (TT)) and scratch the cornea, resulting in corneal opacity and blindness.[2]

The World Health Organization (WHO) strategy for Global Elimination of Blinding Trachoma by the year 2020 (GET2020) is through employment of the SAFE strategy (**S**urgery for trichiasis, **A**ntibiotics for active trachoma, **F**acial cleanliness, and **E**nvironmental improvement).[3] The Gambian National Eye Care Programme (NECP), established in 1986, expanded its national intervention programme to cover the whole country by 1996. NECP activities include the training of health workers in primary eye care, surgery for trichiasis cases, recognition and treatment of conjunctivitis, school screening, and face-washing promotion.[4] The NECP also has a network of *nyateros* (Friends of the Eye) who are non-health professionals identified by their own communities, and have been trained to promote good eye health practices in the community. The programme policy is to treat active trachoma cases and household contacts with tetracycline eye ointment. At the time the survey presented here was conducted (January to March 2006) The Gambia was yet to receive a donation of the antibiotic azithromycin from the International Trachoma Initiative (ITI) for mass treatment.

Evidence from two national surveys carried out in 1986 and 1996 suggested that the prevalence of trachoma fell in The Gambia, with age standardised prevalences of blinding trachomatous corneal opacities falling from 0.10% to 0.02%. In the same time span, the prevalence of active trachoma in 0–14 year-olds fell from 10.4% to 4.9%.[5] Disease prevalence data collected in surveys is of importance to any national control programmes seeking to eliminate blinding trachoma in accordance with the WHO definitions (prevalence of TT less than 1 case per 1000 total population, and prevalence of TF in 1–9 year-old children less than 5%).[3] Data collected in surveys also allow control efforts to be directed to trachoma endemic areas. However, many studies have demonstrated that the prevalence of detected ocular *C. trachomatis* infection is lower than the prevalence of active trachoma, especially in mass treated and low prevalence settings.[6],[7],[8],[9] The Gambia has not collected infection data in its national surveys but these data may lead to a better understanding of the disease's epidemiology with potential implications for the introduction of trachoma control interventions. Mass antibiotic treatment for trachoma is likely to be effective in treating communities with *C. trachomatis* infection, but of questionable value where no infection can be demonstrated. [10],[11]

We aimed to estimate the prevalence of active trachoma and ocular *C. trachomatis* infection in children aged less than 10 years, a decade after the last national survey, in Lower River Region (LRR) and North Bank Region (NBR). These two regions had the highest prevalence of active trachoma in children aged 0–9 years in 1996 (11.5% and 7.7%, respectively).[5] This study reports the results of this cluster-randomised cross-sectional survey in The Gambia, conducted in 2006.

## Methods

### Ethics statement

Research was done in accordance with the declaration of Helsinki. Ethical approval was obtained from the London School of Hygiene and Tropical Medicine (LSHTM), UK, Ethics Committee and The Gambia government/Medical Research Council (MRC) Joint Ethics Committee, The Gambia. Written (thumbprint or signature) informed consent was obtained from the guardians of all children.

### Study design

The estimated populations of NBR and LRR in 2003 were 172,835 and 72,167, respectively.[12] The sample size was constructed for 80% power, with 95% confidence, to detect a region prevalence of TF in 0–9 year olds above 10% if the true prevalence was 12%, or below 10% if the true prevalence was 8%, allowing for the geographical clustering of TF cases by assuming a design effect of 4. The design effect shows the effect of the study design on the estimate's variance, and increases with cluster sample size and within-cluster homogeneity. Since the distribution of trachoma is clustered, and a cluster sampling strategy was employed, a design effect of 4 adjusts the sample size to obtain the same estimate precision as if the disease were homogenously distributed and a simple random sample had been taken.[13],[14]

The survey methods have been described in detail elsewhere.[15] Briefly, a two-stage cluster random sampling strategy with probability of selection proportional to size was employed. Sixty-one enumeration areas (EAs), geographical units of approximately the same population size, were chosen at random from the two regions (42 in NBR and 19 in LRR). EAs are classified in The Gambia's census as rural or urban.[12] A household head list was made for each selected EA and a random selection of households was made by dividing random numbers generated in Excel (MS Excel v2000) with the reciprocal of the number of households in the EA. Households were selected sequentially from the top of this list, duplicates excluded, until 50 children aged under 10 years were included, based on the assumption that the average household size was 7.5 people, of whom about half would be aged 0–9 years. Reserve households were included in case 50 children could not be obtained with the first set number of selected households.

### Field methods

The day before examination, an enumeration team censused the *de facto* population (those who had slept in the household the night before) of the selected households, recording name, alias names, age and sex. The enumeration team identified the children aged under 10 years and informed the households that the examination team would be coming the following day. The examination team examined the children for clinical signs of trachoma in the same order as they had been selected until a total of 50 children per EA was obtained. Two experienced trachoma clinical graders were used, who had successfully achieved a chance corrected agreement (Cohen's kappa statistic [16]) with the standard over the scoring of each sign (TF, TI, TS, TT) of 0.8 or greater in validation exercises with an experienced observer (RLB). These exercises were conducted both in the field and using the WHO trachoma grading slides, and were further supplemented using an in-house slide and photograph collection. Both eyes were graded using a 2.5× magnifying loupe and torchlight. Grading was according to the WHO simplified grading system and the results of the worst eye were reported.[17] In LRR only, ocular Dacron swabs (Hardwood Products Company, Gilford, ME, USA) were taken from the everted tarsal conjunctiva of the child's right eye, using a highly standardised technique.[11] All individuals diagnosed with trichiasis were referred to the nearest health centre for surgery provided free of charge. Individuals with TF or TI were offered treatment with tetracycline eye ointment. Other ocular morbidities were managed according to NECP guidelines.

### Laboratory methods

The swabs were kept cool in the field, frozen within 10 hours, and processed by Amplicor Polymerase Chain Reaction (PCR) assay (Roche Molecular Systems, Branchburg, NJ, USA) at MRC Laboratories, The Gambia. A panel of samples was successfully completed by the laboratory technicians, who had been masked to the sample results.

In order to demonstrate that adequate ocular specimens were taken in the field, all samples were also tested for the presence of human-specific hypervariable 1 (HV1) D-loop region mitochondrial DNA (mtDNA), using D-loop HV1 upper primer L15997, 5′-CAC CAT TAG CAC CCA AAG CT-3′ and D-loop HV1 lower primer H16236, 5′-CTT TGG AGT TGC AGT TGA TG-3′ (Sigma-Genosys, Gillingham, UK).[18] The reaction mixture contained 2 µL of Amplicor extract, 1X Quantitect SYBR Green PCR mastermix (Qiagen, Crawley, UK), each primer at 0.3 µM, and was made up to 10 µL with DEPC-treated sterile water. After denaturation at 95°C for 15 minutes, samples were subjected to 45 cycles of thermal cycling (15 seconds at 95°C, 30 seconds at 60°C and 90 seconds at 72°C) on a Rotor-Gene RG3000 (Qiagen, Crawley, UK). The samples were analysed by melt analysis (72–95°C), which consisted of a single hold/acquisition for 45 seconds at 72°C and then 46 hold/acquisitions at increments of +0.5°C. The HV1 D-loop region amplicon melted at a mean temperature of 81.2°C (range 80.78–81.37, mean standard deviation 0.41). During optimisation, we used gel electrophoresis to confirm the presence of a discrete 278 bp amplicon.

### Statistical analysis

Results were double-entered by different entry clerks and verified in Microsoft Access (MS Access v2000/2003XP). Data cleaning and analyses were performed in Stata (v9.2, STATA Corp., College Station, TX, USA). Any discrepancies after verification and cleaning were checked against the original paper forms. The unadjusted prevalence of TF in 1–9 year-olds is presented as a percentage, as standard practice for a trachoma control programme, at EA, district, and regional levels. At regional level, the design effect and adjusted estimates of prevalence and corresponding 95% confidence bounds were obtained, accounting for the two-stage sampling framework and population size. At EA and district levels, exact binomial confidence intervals were computed around the prevalence, as the study was designed to have an accurate estimate of TF prevalence only at the region level. Associations between the prevalence of TF and districts were tested using the chi-squared (χ^2^) statistic. The spatial distribution of TF prevalence by district and EA was presented graphically using ArcView 3.3 software (Environmental Systems Research Institute, Inc. Redlands, CA, USA).

## Results

Of the 61 randomly selected EAs, 60 consented to participate. A total of 2990 children aged 0–9 years (950 from LRR and 2040 from NBR) were examined from an estimated population of 75,000. Five children from three EAs were excluded from analyses because they were aged over 9 years, and in five EAs only 49 children were screened.

A total of 338 children were enumerated but not examined (Table 1). The age distribution in those examined was significantly different from those not examined (p<0.001), with a higher proportion examined aged 1–5 years and a lower proportion aged 6–9. The sex distribution was similar (p = 0.954). The examined population also differed from the underlying populations of LRR and NBR established in the 2003 census,[12] but in the opposite direction, with a lower proportion examined aged 3–6 and a higher proportion aged 7–9 years. Adjusting for age against the populations established by census led to a TF prevalence in children aged 1–9 years of 13.2% in LRR and 11.3% in NBR, versus 12.3% and 10.0% when unadjusted, respectively. This reflects the peak TF prevalence in children aged 1–5 years.[2] However, since trachoma control programmes usually do not calculate adjusted prevalence rates before making treatment decisions, we have presented unadjusted prevalences.

**Table 1:** Age distribution of children enumerated and children examined, and prevalence of TF, TI and TS in those examined.

Age group (years)	Number of children enumerated	Number of children examined	TF^a^		TI^a^		TS^a^	
			Number of cases	% of children examined (95% CI^b^)	Number of cases	% of children examined (95% CI^b^)	Number of cases	% of children examined (95% CI^b^)
<1	263	213	12	5.6 (2.9–9.6)	0	0 (0–1.7^c^)	0	0 (0–1.7^c^)
1–2	579	542	71	13.1 (10.4–16.2)	2	0.4 (0.04–1.3)	3	0.6 (0.1–1.6)
3–5	851	798	136	17.0 (14,5–19.8)	0	0 (0–0.5^c^)	4	0.5 (0.1–1.3)
6–9	1635	1437	91	6.3 (5.1–7.7)	1	0.1 (0.002–0.4)	17	1.2 (0.7–1.9)
**Total**	**3328**	**2990**	**310**	**10.4 (9.3–11.5)**	**3**	**0.1 (0.02–0.3)**	**24**	**0.8 (0.5–1.1)**

a TF = trachomatous inflammation, follicular; TI = trachomatous inflammation, intense; TS = trachomatous conjunctival scarring.

b Confidence interval.

c One-sided, 97.5% confidence interval.

### Prevalence of clinical signs

Overall, TF was found in 310 (10.4%) children aged 0–9 years (Table 1). Children aged 3–5 years had the highest prevalence of TF (17.0%), followed by those aged 1–2 years (13.1%). Children under one year old and those aged 6–9 years had TF prevalences of 5.6% and 6.3%, respectively. Only three (0.1%) children had TI (two in NBR and 1 in LRR), and two of these also had TF. The left and right eye were concordant for TF (both clinically normal or both with TF) in 2933 (98.1%) children.

**Figure 1:**
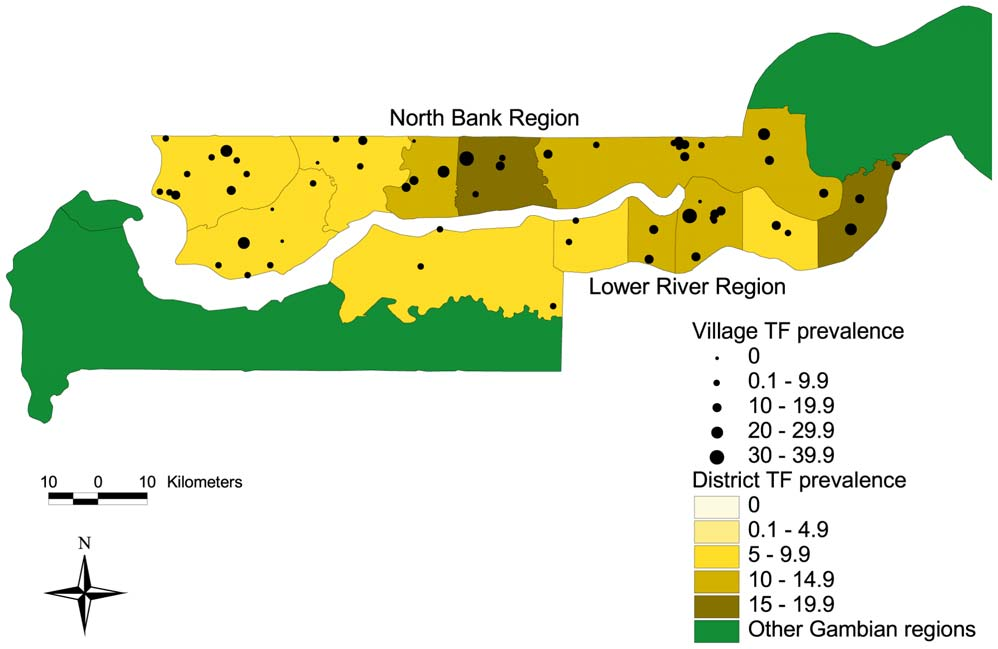
Spatial distribution of TF prevalence in 1–9 year-olds in districts and sampled villages in North Bank and Lower River Regions.

Considering the 1–9 year age group, the overall TF prevalence (the WHO indicator) was 10.7% (95% CI 8.7–13.1). The prevalence of TF in LRR and NBR, respectively, was 12.3% and 10.0% (Table 2). The design effect for heterogeneity among EAs was 3.474 overall; 3.322 for LRR and 3.561 for NBR. The district prevalence varied significantly in LRR (p = 0.006) from 5.7% to 18.2%, and in NBR (p = 0.001) from 5.6% to 15.1%. There was a trend towards higher rates in the eastern districts compared to those in the west in LRR (χ^2^ test for trend p<0.001) but not in NBR (p = 0.287) (Figure 1). Prevalence also appeared to vary dramatically between EAs in the same district. For example, in Jarra West in LRR, prevalence ranged from 0% to 36.7%, and in Central Baddibu in NBR, prevalence ranged from 6.3% to 34.0%. Comparing the results with those from 1996 indicates little change overall: LRR increased slightly from 11.5% to 11.9%, and NBR increased from 7.7% to 9.7%. [5].

**Table 2:** Prevalence of trachomatous inflammation, follicular (TF) in 1–9 year-olds by region, district, and enumeration area (EA).

Region	Examined	TF (%) (95% CId,e)	District	Examined	TF (%, 95% CId,f)	No. of EAs per district	No. of EAs at prevalence (%)				
							0	0.1–9.9	10–19.9	20–29.9	30–39.9
LRRa, c	876	108	Kiang West	140	8 (5.7, 2.5–10.9)	3	0	3	0	0	0
		(12.3)	Kiang Central	95	6 (6.3, 2.4–13.2)	2	0	2	0	0	0
		(8.8–17.0)	Kiang East	94	13 (13.8, 7.6–22.5)	2	0	0	2	0	0
			Jarra West	319	47 (14.7, 11.0–19.1)	7	1	2	3	0	1
			Jarra Central	91	9 (9.9, 4.6–17.9)	2	0	1	1	0	0
			Jarra East	137	25 (18.2, 12.2–25.7)	3	0	0	2	1	0
NBR^b^	1901	190	Lower Niumi	457	39 (8.5, 6.1–11.5)	10	0	7	2	1	0
		(10.0)	Upper Niumi	330	19 (5.6, 3.5–8.8)	7	2	4	0	1	0
		(7.7–12.9)	Jokadu	187	13 (7.0, 3.8–11.6)	4	1	2	0	1	0
			Lower Baddibu	186	27 (14.5, 9.8–20.4)	4	1	0	2	1	0
			Central Baddibu	186	28 (15.1, 10.2–21.0)	4	0	2	1	0	1
			Upper Baddibu	555	64 (11.5, 9.0–14.5)	12	0	5	6	1	0
Total	2777	298 (10.7), (8.7–13.1)		2777	298	60	5	28	19	6	2

a Lower River Region.

b North Bank Region.

c The prevalence of ocular *Chlamydia trachomatis* infection in LRR in children aged 1–9 years and whose ocular swabs tested positive for human mtDNA was 0.3% (3/866); one in Jarra West and two in Kiang East.

d Confidence interval.

e 95% confidence intervals accounting for design effect.

f Binomial exact 95% confidence intervals.

There were three urban EAs in LRR and nine in NBR. Overall, 53 of 552 (9.6%, 95% CI 7.3–12.4) urban children aged 1–9 years had TF, compared with 245 of 2225 rural children (11.0%, 95% CI 9.7–12.4). There was no evidence of variation in TF prevalence between urban and rural EAs in either LRR (p = 0.395) or NBR (p = 0.854).

### Prevalence of ocular *Chlamydia trachomatis* infection

Only 3 of the 950 samples collected in LRR tested positive for ocular *C. trachomatis* by Amplicor. Sample was available for the testing of human-specific hypervariable D-loop region mtDNA in 942 of the 947 Amplicor-negative samples, of which 937 (99.5%) tested positive. Thus, the prevalence of ocular *C. trachomatis* infection in samples positive for human mtDNA was 0.3% (3/940). Three field air controls (swabs waved in the air during fieldwork to control for any field contamination) were also tested for both ocular *C. trachomatis* infection and human mtDNA, giving negative results. The three children who tested positive for ocular *C. trachomatis* infection by Amplicor were aged 9, 8 and 5 years old. One of these was in Jarra West, in an EA with a TF prevalence of 8.0% in 0–9 year-olds, and two were from Kiang East, in an EA with a TF prevalence of 16.0% in 0–9 year-olds. Two Amplicor positive children were clinically normal according to the WHO simplified grading system definition, and the other had bilateral TF. All three Amplicor positives were confirmed positive by a real-time PCR targeting the *ompA* gene.[15]

## Discussion

The results of this survey, covering two regions of The Gambia with a population of approximately 245,000 people, suggest that the overall prevalence of active trachoma in children aged 0–9 years has not noticeably changed since the 1996 national survey. There is some evidence of a secular trend in trachoma prevalence in The Gambia prior to 1996: in one rural village active disease prevalence reduced from the 65.7% documented in 1959 to 2.4% in 1996 in the absence of direct control interventions [19]. However there is no evidence that such a trend continued in LRR and NBR between 1996 and 2006 despite trachoma control efforts being in place in these areas. In contrast to TF, the prevalence of *C. trachomatis* infection was extremely low in this survey, with only 3 of the 940 samples from LRR that were positive for human mtDNA also testing positive for *C. trachomatis*. No infection data were collected in the previous Gambian national surveys so no trends over time can be estimated. In addition, other countries have not collected data on ocular *C. trachomatis* infection when conducting population-based surveys of active trachoma, so between-country comparisons cannot be made. However, our results show a lower prevalence of infection compared with research studies conducted in The Gambia over the last two decades.[10],[20],[21],[22] In 1991, the prevalence of infection among all inhabitants in two Gambian villages was 17.2%.[20] In 1999, the overall prevalence in eight Gambian villages was 35.9%.[21] Burton *et al*. reported an overall prevalence of 7.2% in 14 Gambian villages in 2003,[10] and 19.8% in two villages in 2006.[22] It is important to note that these projects were focused on villages with notable trachoma public health problems usually identified by finding a high prevalence of active trachoma on prior screening, with later laboratory demonstration of high rates of ocular *C. trachomatis* infection. In these villages, therefore, higher prevalence rates may have been recorded than would be estimated in the national Gambian population.

A disparity between the prevalence of active trachoma and of ocular *C. trachomatis* infection has been reported by others, especially in communities where the prevalence of disease is declining, or in communities that have received mass treatment with antibiotics.[6],[9],[10],[20],[23],[24],[25],[26],[27],[28] Solomon *et al.* observed a lag time of several years between infection elimination and the prevalence of TF falling below 5% in a mass-treated Tanzanian community.[7],[26] In a low prevalence region of Nepal, 6.3% of children aged 1–10 years had clinical signs but no *C. trachomatis* was detected.[6] Our results suggest that trachoma may be in decline in The Gambia, with the clearance of clinical signs lagging behind that of infection. That TF cases remain clustered by EA with a design effect of 3.474 may suggest that previous transmission is being captured, or that undetected infections may have existed in close contacts. Despite diagnostic assays within the last decade becoming increasingly more sensitive, the prevalence of detectable *C. trachomatis* infection is now very low. Our observation that the vast majority of cases were TF, with only three cases of TI, supports this hypothesis, as TI cases are more likely to have detectable *C. trachomatis* infection and with a higher organism load.[11],[21],[29],[30] In the one TI case tested here, *C. trachomatis* infection was not detected. Clinically, our impression was that many of the TF cases were rather mild, in the senses that cases with very large numbers of follicles were rarely present, and also that ‘minor signs’ of trachoma such as active pannus and limbal follicles[31], were rare. Possibly therefore increasing the 10% TF threshold for intervention, increasing the number of central zone follicles required to diagnose TF, and including minor signs of trachoma in the assessment, might all improve the ability of clinical signs to predict communities or districts with significant ocular *C. trachomatis* infection. Much more data would however be needed to justify any such departure from the current simplified survey methods.

This association between prevalence of disease, severity of disease, and load of *C. trachomatis* infection could provide another explanation for the observed disparity between prevalence of clinical signs and detected ocular *C. trachomatis* infection. The bacterial load may have been too low for detection by Amplicor PCR. However, the detection limit of Amplicor PCR has been placed at about 2–4 elementary bodies (or 20–40 plasmid copies) per 100 µl,[32] and thus even a few bacteria collected on the swab should have yielded a positive result. The use of a Dacron polyester-tipped swab may have lowered the PCR detection level as it absorbs approximately 200 µl of Amplicor lysis buffer and therefore a higher volume is required to elute the sample. The use of a flocked swab could help overcome this.[33]

In terms of clinical grading, the two examiners were experienced and had fulfilled the criteria of the validation sessions. Swabs were taken according to a standardised protocol [11] by experienced ophthalmic nurses, and 99.5% (937/942) of the samples tested positive for human-specific mtDNA. Thus, we believe that the observed prevalence of active trachoma, as well as the low prevalence of ocular *C. trachomatis* infection, is genuine.

The follicular conjunctivitis (TF) observed in this study could alternatively be due to organisms other than *C. trachomatis*. Potential causes include adenovirus, *Herpes simplex* virus, Epstein Barr virus, *Molluscum contagiosum*, *Moraxella* spp. and other species of *Chlamydia*, such as *C. pneumoniae*, which was first isolated from the eyes of children with trachoma.[6],[34],[35],[36],[37],[38] Further laboratory investigations to test the *C. trachomatis* negative swabs from clinically active children for these other organisms may be warranted.

Our results have two important implications. The first relates to policy decisions regarding control efforts, particularly now that The Gambia has for the first time received a donation of azithromycin for trachoma control. Currently, according to WHO criteria, districts and communities with a TF prevalence greater than or equal to 10% in children aged 1–9 years should receive mass treatment annually, in addition to the ‘F’ and ‘E’ components of the SAFE strategy, for at least three years, until the prevalence of TF falls below 10%.[39] According to these criteria, 3 districts and 10 EAs in LRR, and 3 districts and 17 EAs in NBR, should be treated. However, our sampling strategy was designed to have an accurate estimate of TF prevalence at the region level and not at the community or district level. The size of Gambian regions is similar to the average size of a district in many countries (100,000 to 150,000 people). On this basis, our results suggest that the whole of LRR and NBR should undergo mass treatment according to WHO criteria, as their respective TF prevalence in 1–9 year-olds is 12.3% and 10.0%.

Conversely, the very low prevalence of *C. trachomatis* infection indicates that treatment with antibiotics is almost certainly not necessary in many of these communities, since transmission of *C. trachomatis* is now presumed to be rare. Treatment decisions based on clinical signs of trachoma may lead to unnecessary mass treatment of whole regions or districts, wasting the scarce resources available, although there may be benefits beyond trachoma control to the mass distribution of azithromycin, such as a decline on overall mortality as shown in Ethiopia.[40] A cheap, rapid, point-of-care test that can detect ocular *C. trachomatis* infection could allow policy makers to devise treatment strategies based on the prevalence of infection. At present, no such test is available despite previous encouraging results [41]. Alternatively, the use of laboratory tests, such as PCR, has been suggested as a means of detecting ocular *C. trachomatis* infection. Despite reduced costs through specimen pooling[42], national programmes are unlikely to adopt these assays as they require expensive reagents, electricity-dependent equipment, and highly trained technicians.[43] In the absence of a test for infection for use by national programmes, our observation that TF clustered by EA supports the strategy of treating households containing someone with active trachoma in order to reach infected but clinically normal individuals.[10]

The second implication concerns itself with the question of whether children with clinical signs of trachoma in the absence of *C. trachomatis* infection will develop blinding sequelae. The epidemiology of trachoma is not sufficiently understood to enable this question to be answered for certain, although the evidence that repeated infection is required for disease progression suggests that it is unlikely.[44],[45]

This study shows a large discrepancy between the prevalence of clinical signs and of *C. trachomatis* infection in two Gambian regions in the absence of a national mass treatment programme. The prevalence of TF in children aged less than 10 years in the two Gambian regions that were sampled remains largely unchanged from the prevalence 10 years ago, at around 10%. In contrast, the prevalence of *C. trachomatis* infection is low (0.3%). Our results indicate that The Gambia may not only be on course for certification of blinding trachoma elimination according to the WHO definition (prevalence of TT less than 1 case per 1000 total population, and prevalence of TF in 1–9 year-old children less than 5%)[3], but also on course to eliminate the ocular strains of *C. trachomatis* prior to any mass azithromycin distribution.
